# Modification of the Textural Properties of Chitosan to Obtain Biochars for CO_2_-Capture Processes

**DOI:** 10.3390/polym14235240

**Published:** 2022-12-01

**Authors:** Isabel Barroso-Martín, Juan Antonio Cecilia, Enrique Vilarrasa-García, Daniel Ballesteros-Plata, Carmen Pilar Jiménez-Gómez, Álvaro Vílchez-Cózar, Antonia Infantes-Molina, Enrique Rodríguez-Castellón

**Affiliations:** 1 Departamento de Química Inorgánica, Cristalografía y Mineralogía (Unidad Asociada al ICP-CSIC), Facultad de Ciencias, Campus de Teatinos, Universidad de Málaga, 29071 Málaga, Spain; 2 Grupo de Pesquisa em Separações por Adsorção (GPSA), Departamento de Engenharia Química, Universidade Federal do Ceará, Campus do Pici, Fortaleza 60455-760, CE, Brazil

**Keywords:** chitosan, pyrolysis, biochar, freeze-drying, chitosan-films, CO_2_-adsorption

## Abstract

Three chitosans with different morphologies have been used (commercial chitosan powder, chitosan in film form and chitosan in globular form synthesized by the freeze-dried method) for the synthesis of biochars. The pyrolytic treatment has revealed that the biochar synthesized from the chitosan formed by the freeze-dried method reaches the highest CO_2_-adsorption capacity (4.11 mmol/g at 0 °C and a pressure of 1 bar) due to this adsorbent is highly microporous. Moreover, this biochar is more resistant to the pyrolytic treatment in comparison to the biochars obtained from the commercial chitosan and chitosan in the form of film. CO_2_-adsorption studies at different temperatures have also shown that the adsorption capacity diminishes as the adsorption temperature increases, thus suggesting that the adsorption takes place by a physical process.

## 1. Introduction

In recent decades, the increase in world population and resource consumption has led to an increase in anthropogenic CO_2_ emissions. The increase in the concentration of CO_2_ in the atmosphere has caused serious damage from an environmental point of view and the presence of this atmospheric-CO_2_ limits the release of heat from the planet, causing a phenomenon called the greenhouse effect. This phenomenon produces an increase in temperature on the earth’s surface with serious environmental consequences. Among the main consequences of the greenhouse effect, the melting of glaciers, flooding of islands and coastal cities, migration of species, desertification of fertile areas, and impact on agriculture and livestock can be highlighted. In addition, it also affects human health and causes food shortages, the spread of diseases and pandemics. Considering these devastating effects of global warming, governments have become aware of how to minimize CO_2_ emissions. Nowadays, dependence on fossil fuels is very high, so achieving zero-CO_2_ emission processes is very complex. As an alternative to fossil fuels, renewable energies have been developing in recent decades, although they are not mature enough to provide the demands of the planet actually. Other alternatives are focused on the design of more efficient processes where CO_2_ emissions can be minimized.

Currently, there is no sufficiently mature technology to mitigate CO_2_ emissions into the atmosphere, so a set of complementary measures is required to reduce CO_2_ emissions. One of the most advanced technologies are CO_2_ capture and storage (CCS) and CO_2_ capture and utilization (CCU) [1]. One of the most expensive steps in both processes is CO_2_ capture, so it is necessary to search for and develop low-cost adsorbents to make these processes competitive and sustainable [2,3,4]. Among the low-cost solvents studied in CO_2_ capture processes, the high adsorption values of alkaline earth oxides can be highlighted, although these materials have the disadvantage of their high energy cost in their regeneration stage between 500 and 800 °C [5,6]. Other low cost and high potential adsorbents in CO_2_-capture processes are clay minerals [7,8]. These clays, formed by aluminosilicates, can host CO_2_ molecules on both their surface and between layers, although the clay minerals that present higher CO_2_ adsorption values are the fibrous clays, which present nanocavities in their structure [8,9]. In addition, an advantage of the clays is related to their ability to modify their textural properties by inserting pillars [10,11,12] or acid treatments to increase its adsorption capacity [8,9]. Some clay minerals can also give rise to zeolites by an inexpensive hydrothermal treatment in a basic medium [13,14,15].

Biochar and activated carbons are other interesting materials for CO_2_ capture, which can be considered as low-cost adsorbents if the starting material is inexpensive [16,17]. Several studies have used as a starting material lignocellulose biomass due to this source being highly available on Earth and is an unvaluable agricultural waste in many cases [17].

After cellulose, chitin is the second most abundant biopolymer in the world, with a production of 100 billion Tons/year, of which around of 70% comes from marine sources [18]. Chitin, poly(β-(1-4)-*N*-acetyl-D-glucosamine), is a biopolymer that appears mainly in the exoskeleton of crustaceans and mollusks but can also appear in insect cuticles or fungi [18]. Despite its high abundance and good thermal stability, the main drawback of this biopolymer is related to its low solubility in H_2_O, limiting the number of applications [18,19]. However, chitin can be deacetylated through an alkaline treatment to obtain chitosan poly(β-(1-4)-D-glucosamine) [20], which is highly soluble in mild acid solutions [21], obtaining an inexpensive material with interesting physicochemical properties in such a way that chitosan is applied in pharmacology, medicine as biomaterials, drug delivery, wound, burns, and blood vessel as main applications [22]. It can also be employed in agricultural, antimicrobial applications, as well as in the adsorption of pigments and dies or in catalytic reactions [22].

In the present research, chitosan was selected as a biopolymer to be pyrolyzed to obtain a biochar. In this sense, it has been reported that the biochar obtained from cellulose leads to materials with narrow pore diameters, which allow for host CO_2_ molecules [17]. Considering that chitosan and cellulose display a similar chemical composition where an -OH group is replaced by a -NH_2_ group in chitosan, it is expected that the biochar obtained can reach a good CO_2_-adsorption capacity. The presence of -NH_2_ groups in chitosan also allows for the presence of N-species in the obtained biochar. In this way, it is well-known that the presence of N-species promotes the chemical adsorption of the CO_2_, although the efficiency of this process is more favored for primary amines, as was studied in porous silicas [23,24,25].

Previous studies have reported that the biochar or activated carbons obtained from chitosan by chemical activation or functionalized by amine groups reach a high CO_2_ adsorption capacity and a good selective adsorption in comparison to other gases such as N_2_ [26,27,28], which makes these carbons very interesting to be used for selective adsorption in flue gas. The aim of the present research is to study how the morphology of the chitosan influences the synthesis of biochar and its textural properties as well as the CO_2_-adsorption capacity without additional modifications to avoid an increase in cost-production. For this purpose, chitosans with three morphologies were studied: Commercial chitosan powder, film of chitosan solubilized at acid pH and freeze-dried chitosan, which was previously solubilized.

## 2. Materials and Methods

### 2.1. Reagents

Biochars were synthesized from a chitosan (50,000–190,000 kDa, Aldrich, St. Louis, MO, USA). Acetic acid (99%, VWR, Radnor, PA, USA) was employed to dissolve chitosan in acid medium. The gases used in the adsorption studies were CO_2_ (99.999%, Air Liquide, Paris, France), N_2_ (99.9999%, Air Liquide, Paris, France) and He (99.999%, Air Liquide, Paris, France).

### 2.2. Synthesis of the Adsorbents

For the synthesis of the biochars obtained from chitosan, three approaches were carried out. The first type of chitosan used for pyrolysis was a commercial chitosan used directly. The second type of chitosan was obtained from the dissolution of the commercial chitosan in acid medium. In this synthesis, 2 g of the commercial chitosan were suspended in 200 mL of H_2_O. Then, 1 mL of acetic acid was added to dissolve chitosan, obtaining a gel. In these conditions, the -NH_2_ group of chitosan was transformed into -NH_3_^+^, promoting its dissolution in aqueous medium.

**Figure 1:**
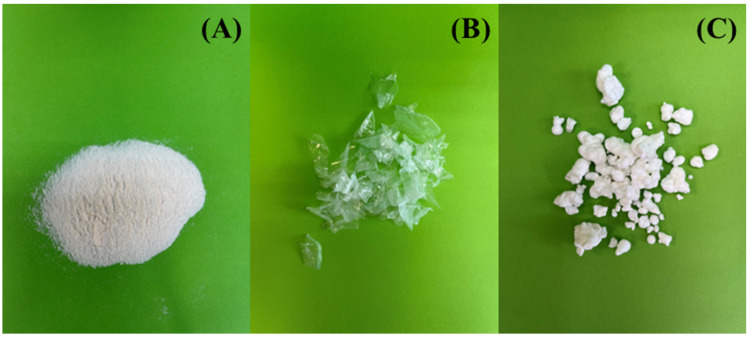
Images for the precursors of Chi-P (**A**), Chi-F (**B**), and Chi-G (**C**).

Finally, the gel was transferred and dried at 50 °C overnight to evaporate the H_2_O of the solution, obtaining a chitosan in the form of film. The third type of chitosan followed the same procedure as the second. Commercial chitosan was suspended in H_2_O and dissolved in the same amount of acetic acid. Then, the obtained gel was freeze-dried to remove the H_2_O of the solution, obtaining chitosan with a globular form. The structures of the obtained chitosans are compiled in Figure 1, while the scheme of the chitosan solubility is included in Figure S1 of the Supplementary Materials.

In the next step, chitosans with different morphologies were pyrolized in N_2_-medium at different temperatures to obtain the corresponding biochars.

The synthesized biochars were labeled as Chi-X-Y, where X is the morphology of the starting chitosan (P, powder for the raw chitosan; F, film for the lamellar chitosan; G, globular for freeze-dried chitosan). The term Y represents the temperature of the pyrolytic treatment.

### 2.3. Characterization of the Adsorbents

The crystallinity of the biochars was studied by X-ray diffraction (XRD). The diffractograms were collected in an Empyrean PANalytical diffractometer (Malvern, UK) (Cu Kα_1,2_) equipped with a PIXcel 1D detector in a Bragg Brentano configuration between 2θ of 10° and 80° using a step size of 0.026° and a counting time of 24 s/step. The diffractometers were carried out with a voltage of 40 V and a current of 30 A at room temperature.

The morphology of the chitosans and the biochars obtained after the pyrolytic treatment at 900 °C was studied using a scanning electron microscope (SEM) with JEOL SM-6490 LV equipment (Tokyo, Japan). The samples for SEM observation were gold-sputtered to avoid charging of the surface.

^13^C-NMR spectra were collected at room temperature using AVANCEIII HD 600 (Bruker AXS) equipment (Billerica, MA, USA) using a triple resonance CP-MAS probe of 3.2 mm at a spinning rate of 15 kHz. The magnetic field is 14.1 T, corresponding to a ^13^C resonance frequency of 150.91 MHz. The spectra were recorded with 7-s delay with ^1^H decoupling (^13^C Hpdec with tppm15 decoupling sequence for C) and summing up 5000 scans. The ^13^C chemical shifts are referenced to adamantane.

Raman experiments were performed in a micro-Raman JASCO NRS-5100 spectrometer (Easton, PA, USA). This equipment combines a dispersive Raman spectrometer and a confocal microscope, equipped with DSF (Dual Spatial Filter) to diminish optical aberrations and to improve the resolution up to 0.4 cm^−1^. The calibration of the different laser lines was made with respect to silicon, while the detection was achieved by means of a high-resolution CCD (Charge Coupled Device). All the experiments were carried out using a Nd:YAG laser with a wavelength of 532 nm, a power of 2.4–4.6 mW, an acquisition time of 10 s with 10 accumulations and an objective of 100X.

Thermal analysis was carried out on a TG-DTG thermobalance (Mettler Toledo) (Columbus, OH, USA) with a continuous heating rate of 5 °C/min in N_2_-flow 50 mL/min from 30 to 900 °C.

The chemical composition of the biochars on the surface was performed by X-ray photoelectron spectra. The experiments were carried out using a Physical Electronic PHI 5700 spectrometer (Physical Electronics) (Chanhassen, MN, USA), which is equipped with an Electronics 80–365B multichannel hemispherical electron analyzer and a Mg Kα X-ray excitation source (300 W, 15 kV, hv = 1253.6 eV). High-resolution spectra were recorded by a concentric hemispherical analyzer in a constant energy mode of 29.35 eV, using an analysis area with a diameter of 720 μm. The pressure in the analysis chamber was kept below 5 × 10^−6^ Pa. Binding energies were determined to an accuracy of ±0.1 eV, using the adventitious carbon C 1*s* signal, located at 284.8 eV, as reference. A PHI ACCESS ESCA-F V6 software was used for data acquisition and analysis. A Shirley-type background was subtracted from the signals. The recorded spectra were always analyzed with Gauss-Lorentz curves.

The N-content of the biochars obtained after the pyrolytic treatment of chitosan with different morphologies was determined by elemental chemical analysis using a CHNS 932 analyzer supplied by LECO (Geleen, The Netherlands) through the combustion of the samples at 1100 °C in pure O_2_.

The textural properties of the biochars were determined from CO_2_ adsorption isotherms at 0 °C using a Micromeritics 2420 apparatus (Norcross, GA, USA). Prior the adsorption experiments, the samples were outgased at 120 °C overnight. After the adsorption isotherms, both surface area and micropore volume were determined using the Dubinin-Astakhov equation [29].

### 2.4. CO_2_ Adsorption Isotherms

To evaluate the potential of the synthesized biochars in CO_2_ adsorption processes, the samples were outgased at 120 °C overnight, and then the adsorbents were studied at 0, 25 and 45 °C, between 0 and 760 mm of Hg in a Micromeritics 2420 equipment (Norcross, GA, USA).

### 2.5. Adsorption Models

The empirical Toth equation [30] was employed to describe the adsorption equilibrium. The advantage of this model is that, at low pressures, the Toth equation (Equation (1)) reduces to Henrýs law. The Toth equation can be written as follows:

(1) qi=qmi(biPi)(1+(biPi)ti)1ti

The limiting adsorbed concentration $q_{m}$, the affinity parameter *b* and the heterogeneity factor *t* were varied with the isotherm temperature according to the empiric Equations (2)–(4), respectively.

(2) qmi=qmoi+Xi(1T−1T0)

(3) bi=boiexp[QR (1T−1T0)]

(4) t=toi+ki(1T−1T0)

To estimate the accuracy of each fit, the Average Relative Error (ARE) (Equations (5)), was used:

(5) $$ARE\left(\%\right)=\frac{1}{N_{T}}\sum_{i=1}^{N_{T}}\frac{|q_{i,exp}-q_{i,est}|}{q_{i,exp}}\times 100\;$$

where *N_T_* is the total number of the data points, and *q_i,est_* and *q_i,exp_* are the estimated and experimental amounts of CO_2_ adsorbed, respectively.

## 3. Characterization of the Adsorbents

**Figure 2:**
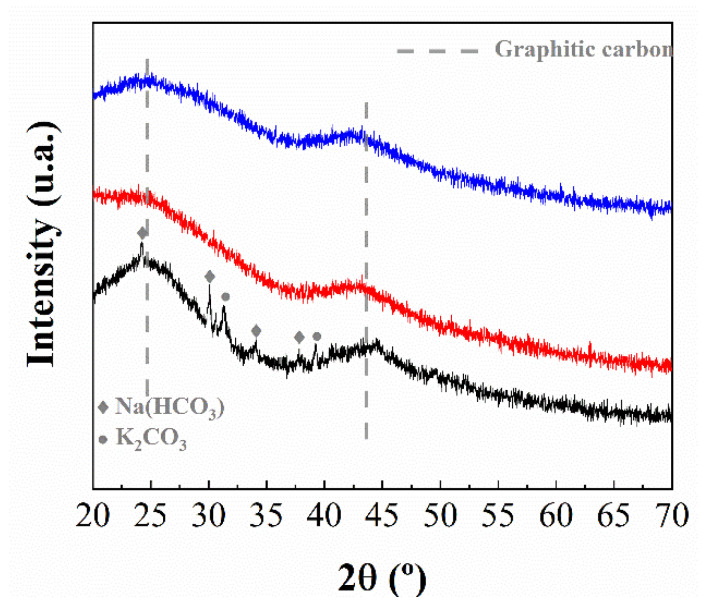
X-ray diffraction of the chitosan with different textural properties pyrolyzed at 900 °C: Chi-P-900 (black), Chi-F-900 (red) and Chi-G-900 (blue).

The crystallinity of the biochars synthesized from chitosan was studied by XRD (Figure 2). With the exception of the sample synthesized from the commercial chitosan (Chi-P-900), all diffractograms display two broad diffraction peaks located at about 2θ of 25 and 44°, which are assigned to the presence of a graphitic carbon with low crystallinity (PDF N: 41-1487) [31]. In the case of the Chi-P-900, narrow but weak diffraction peaks can also be observed, which are ascribed to carbonates and/or hydrogen carbonates (PDF N: 00-025-0626) of K or Na (PDF N: 01-074-1203). These alkaline species must be ascribed to the purification treatment of the crustacean shells as well as to the deacetylation process of chitin to form chitosan [32].

**Figure 3:**
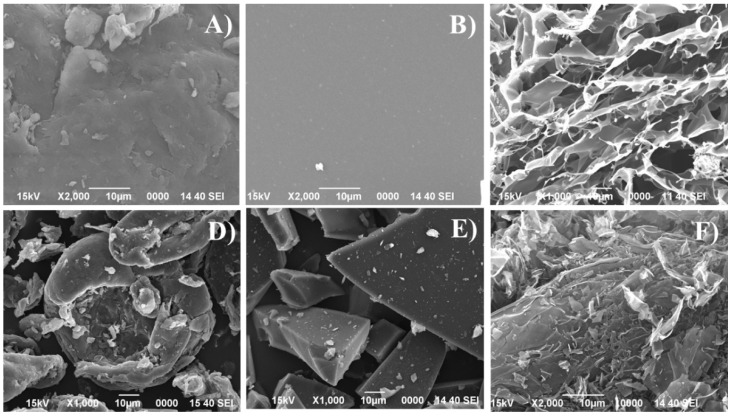
SEM images of Chi-P (**A**), Chi-F (**B**), Chi-G (**C**) and their respective samples pyrolyzed at 900 °C: Chi-P-900 (**D**), Chi-F-900 (**E**) and Chi-G-900 (**F**). (Scale: 10 µm).

The morphology of the biochars obtained from the pyrolysis of chitosan with different treatments was determined by SEM (Figure 3). From these images, how all starting chitosans, prior to the pyrolysis, display different morphologies can be observed (Figure 3A–C).

In the case of commercial chitosan without treatment (Chi-P), the image with lower magnification (Figure S2, Supplementary Materials) shows an anisotropic structure where the dimensions with the width and length of the particles range between 50 and 150 µm. The increase in the magnification of the Chi-P sample (Figure 3A) reveals that these particles are formed by a set of layers stacked on each other, because of the electrostatic interactions with adjacent sheets. The treatment of chitosan in an acidic medium to solubilize it and its deposition in a crystallizer until the solution evaporates results in a layered chitosan without imperfections (Figure 3B). When this dissolved chitosan is subjected to a freeze-drying process instead of being dried in a crystallized process, how the morphology clearly changes can be seen (Figure 3C). This image shows how the globular structure displays many cavities due to the loss of H_2_O in the lyophilization process.

After pyrolytic treatment of each sample at 900 °C, a change in morphology can be observed in comparison to their respective starting materials due to the thermal treatment causing a partial shrinkage and collapse. Thus, the pyrolyzed commercial chitosan shows a partial shrinkage of the particles, whose size is below 100 µm (Figure 3D and Figure S2, Supplementary Materials). In the same way, the structure of the chitosan in the form of a film also collapses after the pyrolytic treatment at 900 °C (Figure 3E), obtaining lamellar but highly fractured structures. In the case of chitosan obtained by the freeze-drying method, pyrolysis at 900 °C also causes a collapse of the structure (Figure 3F), diminishing the dimensions of the cavities.

**Figure 4:**
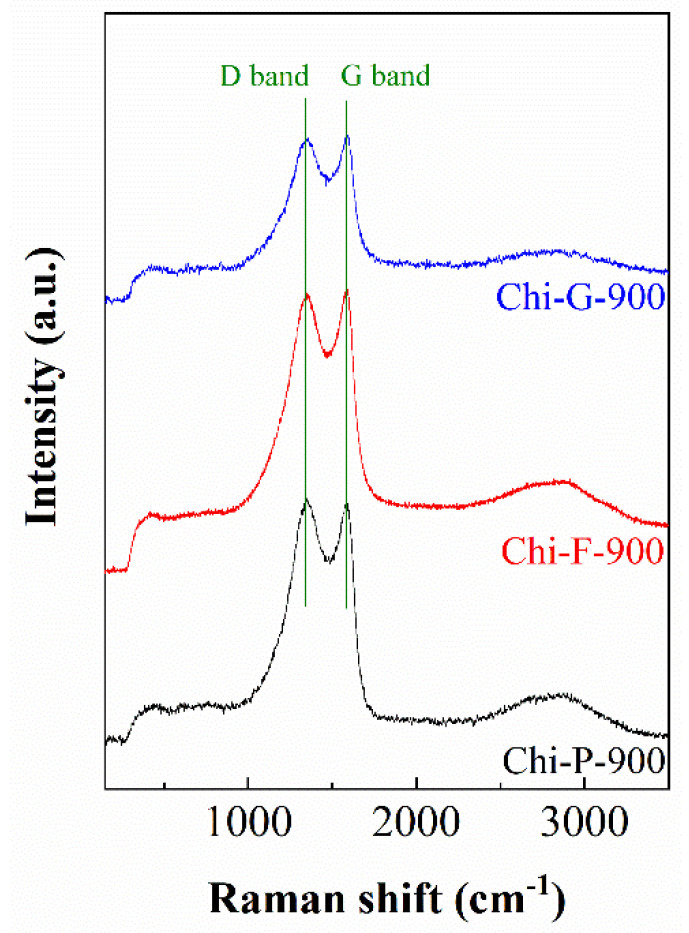
Raman spectra of biochars obtained from chitosans with different morphologies pyrolyzed at 900 °C.

The biochars obtained from the pyrolysis of chitosan were also studied by Raman spectroscopy (Figure 4). In the spectra of the three synthesized materials, two defined bands can be observed, which are located at 1340 and 1590 cm^−1^. These bands are denoted in the literature as D (disorder) band and G (graphite) band, respectively [17,33]. These authors pointed out that the D band is related to the A_1g_ vibration mode, which is like an in-plane breathing vibration type; however, this vibration mode is inactive in a large single crystal-like highly oriented pyrolytic graphite [33]. Regarding the G band, this signal is ascribed to the Raman active E_2g_ in-plane vibration mode [33].

The biochars obtained from chitosan pyrolysis were also studied by Raman spectroscopy (Figure 4). In the three synthesized materials, two defined bands can be observed, which are located at 1340 and 1590 cm^−1^. These bands are denoted in the literature as the D band (disorder) and G band (graphite), respectively [17,33]. These authors pointed out that the D band is related to the A_1g_ vibrational mode, which is like an in-plane breathing type of vibration; however, this vibrational mode is inactive in a large single crystal such as highly oriented pyrolytic graphite [33]. With respect to the G band, this signal is attributed to the Raman active E_2g_ in-plane vibration mode [33].

Considering these premises, it is possible to know the type of carbonaceous material obtained after the pyrolytic treatment. Thus, it has been reported in the literature that an increase in temperature in the pyrolytic treatment causes a progressive decrease in the D band [17,34]. As this band is inactive considering that a graphitic structure is formed, it is expected that the increase in temperature leads to graphitic materials although these materials must be amorphous, or the crystallinity must be very low, as suggested by XRD (Figure 2).

From the Raman data, reported in Figure 4, it can be observed how the maximum values of the D and G bands are similar. These intensities agree to those obtained for other biochars obtained from cellulose at 900 °C [17] so the type of biopolymer (polysaccharide) and its morphology do not seem to play a relevant role in the formation of the graphitic adsorbent, but rather the temperature is the key parameter for the formation of the adsorbent.

**Figure 5:**
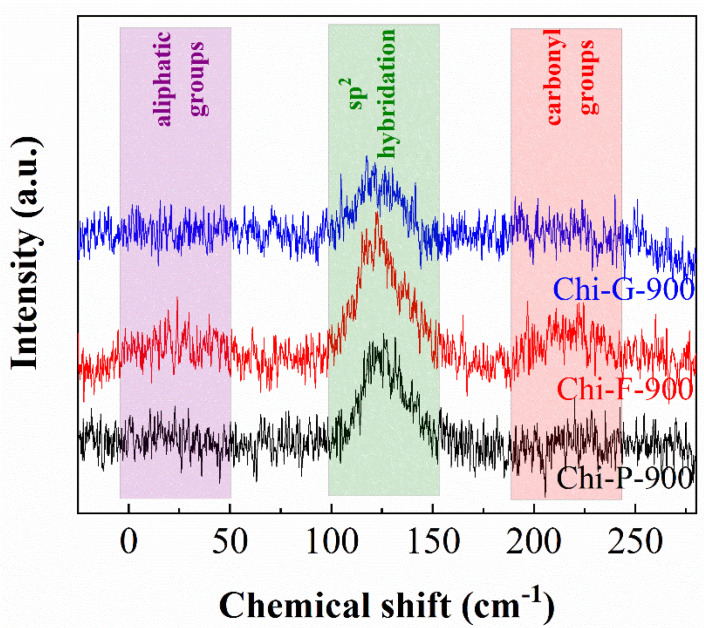
^13^C NMR of biochars obtained from chitosans with different morphologies pyrolyzed at 900 °C.

The synthesized biochars were also characterized by ^13^C NMR (Figure 5). Three well-defined regions can be observed, mainly in the case of biochar Chi-F-900. The main signal, whose maximum is located around 125 ppm, is assigned to the presence of sp^2^ hybridization, thus confirming the formation of a graphitic structure [17,35]. In the same way, two less intense signals can also be observed, which are located between 0–50 and 190–240 ppm [17]. These signals are assigned to the presence of aliphatic and carbonyl groups, respectively.

The analysis of the textural properties was determined from CO_2_ adsorption-desorption isotherms at 0 °C. Many studies have analyzed the textural properties using N_2_ as a molecular probe. However, this molecule is not suitable for poorly accessible pores since it is difficult to reach equilibrium conditions.

**Figure 6:**
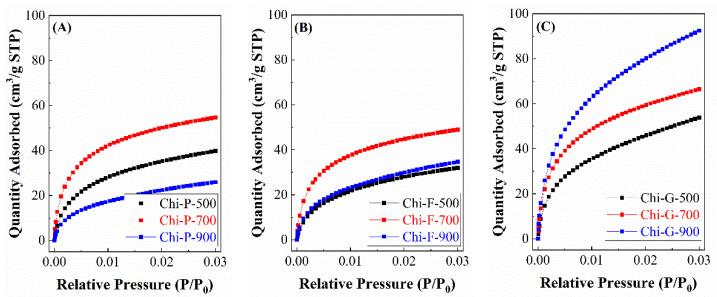
CO_2_ adsorption isotherms at 0 °C for the biochars obtained from the chitosans with different morphologies. (**A**) biochars obtained form chitosan directly, (**B**) biochars obtained from chitosan in the form of film and (**C**) biochars obtained from chitosan in globular form.

Thus, the CO_2_ adsorption isotherms at 0 °C (Figure 6) shows how the pyrolysis of commercial chitosan (Chi-P) increases the CO_2_ adsorption when the temperature of the pyrolytic treatment increases, obtaining the maximum value when the treatment takes place at 700 °C. At higher temperatures, it is striking how the adsorption capacity becomes practically negligible, probably due to the collapse of the carbonaceous structure, giving rise to a material with a very low microporosity. In the case of biochars derived from the pyrolysis of chitosan films (Chi-F), the trend of CO_2_ adsorption is like commercial chitosan, i.e., CO_2_ adsorption increases with the temperature of pyrolysis, obtaining the maximum adsorption value at 700 °C, and at higher temperatures, the adsorption capacity decreases, although to a lesser extent than for commercial chitosan. Finally, the chitosan treated by the freeze-drying method (Chi-G) shows an increase in CO_2_ adsorption as the pyrolytic treatment increases.

Therefore, these data evidence that, although all the materials display a similar chemical composition (amorphous graphite), the adsorption capacity of the different biochars depends on both the staring chitosan and the pyrolytic temperature employed.

The determination of the microtextural properties was determined from the CO_2_ adsorption isotherms at 0 °C, using the Dubinin-Astakhov equation (Table 1) [29]. From these data, it can be observed that the higher microporosity is attained for those biochars synthesized from chitosan modified by the freeze-drying method, reaching a maximum micropore volume of 0.263 cm^3^/g and a surface area of 657 m^2^/g for the sample pyrolyzed at 900 °C (Chi-G-900). Commercial chitosan (Chi-P) displays higher porosity than chitosan in the form of film (Chi-F). In both cases, the maximum microporosity is obtained at 700 °C (0.158 cm^3^/g for Chi-P-700 and 0.148 cm^3^/g for Chi-F-700). At higher temperatures, the structure of commercial and film chitosan collapse, although this collapse is more pronounced in the case of commercial chitosan and is related to the loss of microporosity in the resultant biochar. In any case, the textural properties of the solids obtained are higher than the other solids reported in the literature [36,37,38,39].

**Figure 7:**
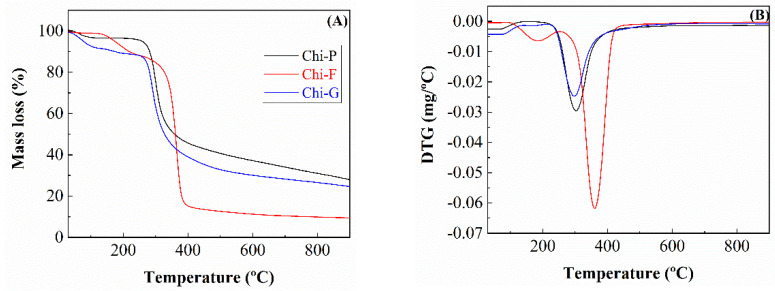
Thermogravimetric (**A**) and differential thermal analysis (**B**) of Chi-P, Chi-F and Chi-G precursors.

Considering that the textural properties of the pyrolyzed chitosan depend on the starting morphology (Figure 3 and Figure 6), the next characterization study was the analysis of the thermogravimetric loss using an inert gas such as N_2_ (Figure 7). Figure 7 shows how the profiles for the synthesis of Chi-P and Chi-F adsorbents are very similar between them. In both cases, the physisorbed-H_2_O is removed until 250 °C, although the content is different since the Chi-G-900 sample loses about 13% of the H_2_O, while commercial chitosan (Chi-P-900 sample) hardly losses 6% of its starting weight. From 250 °C, a pronounced loss in the mass of chitosan because of the pyrolysis takes place until 350 °C. Then, at higher temperatures, the mass loss is more progressive, remaining a mass of 28% for Chi-P and 24% for Chi-G at 900 °C, respectively. In the case of Chi-F, the TG analysis shows a profile that differs from those shown for the Chi-P and Chi-G samples. Thus, the Chi-F sample seems to be stable up to 150 °C. Then, the physisorbed-H_2_O is released between 150 and 290 °C. From this temperature, the TG analysis shows a drastic mass loss between 320 and 400 °C. Then, the Chi-F sample hardly losses mass at higher temperatures. It should be noted that the adsorbents synthesized from chitosan films (Chi-F) are those that have a greater mass loss, as shown in the TG data, where the remaining mass at 900 °C is less than 10% compared to its initial weight. From these data, it can be inferred that, despite all the materials having the same chemical composition, the morphology seems to have an important role in the pyrolytic treatment and in the textural properties obtained where the biochar synthesized from chitosan in the form of film is the material that loses the most mass, breaking down its structure, as was observed from Figure 3.

**Figure 8:**
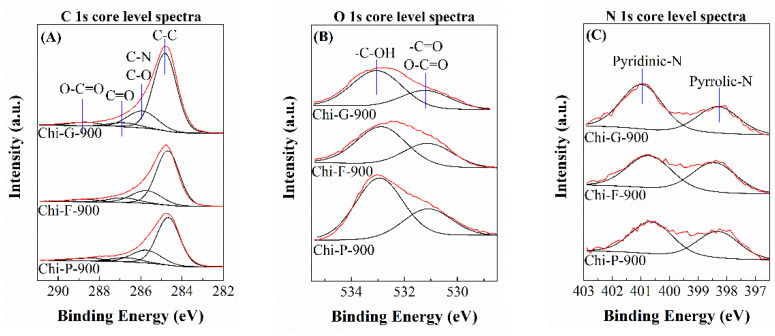
C 1s (**A**), O 1s (**B**) and N 1s (**C**) core level spectra of the biochars obtained after the pyrolytic treatment of chitosans with different textural properties at 900 °C.

The surface chemical composition, as well as the chemical states of the elements on the surface, were determined by XPS (Table 2 and Figure 8). The analysis of the biochars pyrolyzed at 900 °C mainly shows the presence of C, N and O. The analysis of the C 1*s* core level spectra of the biochars (Figure 8A) displays four contributions. In all cases, a main band located at 284.8 eV can be observed, which is assigned to the adventitious carbon or C-C bonds. In addition, the second contribution located about 286.0 eV is attributed to the C-N or C-O groups, thus confirming that O and N-species are present in the carbonaceous structure in the form of amorphous graphite. At higher binding energies, approximately 287.0 eV, other small contributions assigned to C=O can be observed, while the band located at higher binding energy in this region (289.0 eV) is attributed to the presence of the O-C=O groups [40,41].

In the analysis of the O 1*s* region (Figure 8B), two regions can be observed in all cases. The contribution located at lower binding energy, about 531 eV, is assigned to the presence of remaining -C=O groups, while the contribution located at higher binding energy value (533 eV) is assigned to -C-OH groups.

Regarding the N 1*s* core level spectra of the biochars (Figure 8C), all spectra display two contributions. The binding energy located at a lower value (398.5 eV) is assigned to pyridinic-N, while the contribution at around 400.6–400.9 eV is assigned to pyrrolic-N [42], confirming that N-species are embedded within the graphitic structure.

The surface atomic concentration of the biochar was also studied (Table 2). As was expected, the main element in all cases is carbon with an atomic concentration between 80–90%. The oxygen content is between 6–12%, while the nitrogen content is between 3–5%. These values are below the values obtained by CHN where the N-content is 6.4% for Chi-P-900, 7.5% for Chi-F-900 and 5.4% for Chi-G-900, respectively, so a portion of the N-species must not be exposed on the surface of the adsorbents. From XPS data, it can be concluded that the pyrolytic treatment does not completely remove oxygen and nitrogen species. In fact, it seems that nitrogen is incorporated within the structure of amorphous graphite.

## 4. Adsorption Studies

Once the biochars synthesized from a pyrolytic treatment of chitosans with different textural properties were characterized, these materials were tested as potential adsorbents in CO_2_ capture processes.

**Figure 9:**
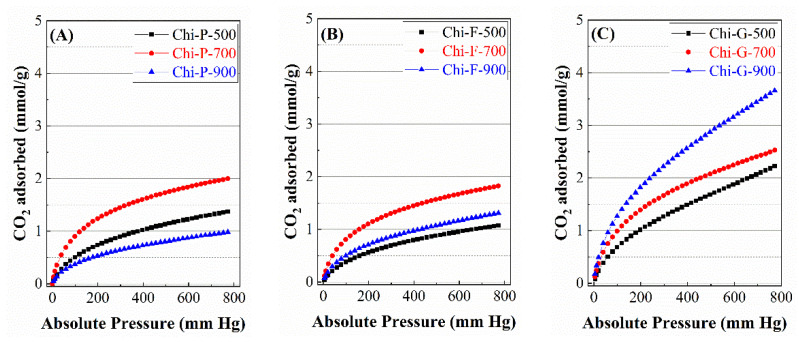
CO_2_ adsorption isotherms at 25 °C and 1 bar of the biochars obtained after the pyrolytic treatment of chitosans with different textural properties at 900 °C. (**A**) biochars obtained form chitosan directly, (**B**) biochars obtained from chitosan in the form of film and (**C**) biochars obtained from chitosan in globular form.

In the first study, biochars synthesized at different temperatures were studied (Figure 9). The biochars synthesized from commercial chitosan (Chi-P) (Figure 9A) show how CO_2_ adsorption is very poor for the adsorbent synthesized at 500 °C (Chi-P-500) since the adsorption capacity is 1.37 mmol/g at a pressure of 1 bar and 25 °C. The increase in temperature in the pyrolytic treatment causes an improvement in microporosity, as indicated by studies of textural properties (Table 1). This means that the biochar can retain a greater amount of CO_2_ in its microporous structure, achieving a capture of CO_2_ of 2.01 mmol/g at 25 °C and a pressure of 1 bar for Chi-P-700 sample. An increase in the pyrolysis temperature to 900 °C promotes the microporosity of biochars by physical activation. However, the textural properties (Table 1) and the adsorption capacity decrease drastically, reaching a CO_2_ adsorption capacity of only 0.97 mmol/g at 25 °C and 1 bar of pressure. This decrease must be ascribed to the fact that the increase in porosity at 900 °C must cause a partial collapse of the porous structure, thus impoverishing its textural properties.

When chitosan is dissolved in an acid medium, it is deposited to obtain a film that is finally pyrolyzed (Figure 9B), the CO_2_ adsorption capacity worsens compared to biochar obtained from commercial chitosan (Figure 9A), probably due to the formation of the film takes place at a stacking of chitosan sheets that can limit the physical activation in the pyrolytic treatment. Thus, the sample pyrolyzed at 500 °C (Chi-F-500) reaches a CO_2_ adsorption capacity of 1.07 mmol/g at 25 °C and 1 bar of pressure. The increased pyrolytic treatment also improves the porosity and 1.82 mmol/g at 25 °C and 1 bar of pressure. As was observed for commercial chitosan pyrolyzed at 900 °C, the pyrolysis at the highest temperature worsens the adsorption capacity (1.31 mmol/g), although this decrease is less pronounced than that observed in commercial chitosan, so that chitosan in the form of film seems to be more stable and resistant to pyrolytic treatment, as was observed from the analysis of the textural properties (Table 2).

The study of the CO_2_ adsorption capacity for the adsorbents synthesized from modified chitosan by freeze-drying (Figure 9C) shows the highest adsorption in the range of the studied pyrolysis temperature. These values must be ascribed to the prior treatment of the chitosan since its dissolution, and then, the freeze-drying method favors the removal of H_2_O, generating a chitosan structure with a high porosity, as was observed in Figure 3C. Thus, the pyrolytic treatment at 500 °C achieves an adsorption capacity of 2.18 mmol/g. The adsorption capacity improves at 700 °C, attaining a value of 2.53 mmol/g. However, the most striking result is obtained when this chitosan is pyrolyzed at 900 °C since, unlike adsorbents obtained from commercial chitosan or in the form of film, the treatment at the highest temperature improves the adsorption capacity, reaching the highest CO_2_ capture with a value of 3.64 mmol/g. These data suggest that the chitosan synthesized from the dissolution and its freeze-drying leads to a material with higher porosity and higher thermal stability, which is more resistant to a collapse in its structure at high temperature. However, it should be noted that these values are above those obtained by biochar from agricultural residues, where a CO_2_ adsorption of 2.63 mmol/g is obtained for a biochar obtained at 900 °C under similar synthetic and analytic conditions [43].

**Figure 10:**
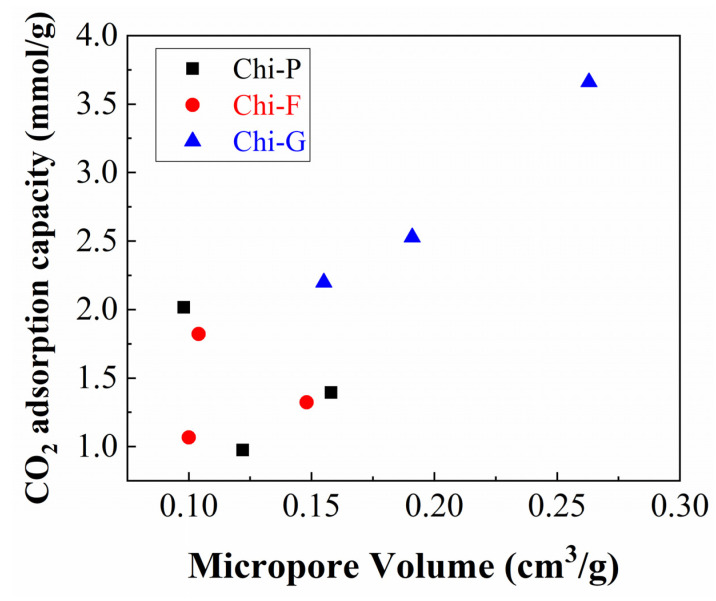
Representation of the CO_2_ adsorption capacity at 1 bar and 25 °C vs. micropore volume, determined from the CO_2_ adsorption isotherm at 0 °C.

From the CO_2_ adsorption isotherms obtained at different temperatures of pyrolysis (Figure 9), it can be concluded that the CO_2_ adsorption capacity is directly related to the microporosity of the biochar, as shown in the linearity between micropore volume vs. CO_2_ adsorption capacity (Figure 10).

As the Chi-G-900 adsorbent reached the highest adsorption capacity, those adsorbents synthesized by the pyrolytic treatment at 900 °C were fitted to the Toth model [30] (Table 3).

In all cases, it can be observed how all the isotherms fit the Toth model well, as indicated by the low ARE values, which are close to zero. Regarding the maximum adsorption capacity when the isotherm is extrapolated to infinite pressure (*q*_*m*0,*i*_), it can be observed that the higher *q*_*mi*,0_ is obtained for the Chi-G-900 sample, which reaches a value of 18.23 mol/kg. These values are well above those obtained for the other biochars synthesized from chitosans with other textural properties due to the greater microporosity of this adsorbent. The greater microporosity of the Chi-G-900 sample supposes a stronger interaction and affinity between the adsorbent and the CO_2_ molecules, as indicated by the *b*_0,*i*_ parameter as well as a higher adsorption heat value, as shown in the Q value. However, the heterogeneity of all adsorbents is very similar among them. These values are far from the unity where all the centers would be equally accessible and available. The *t*_0,*i*_ values are in the range of 0.25–0.28. This implies that there must be active sites that are more likely to capture CO_2_. These sites must be the narrowest pores where the CO_2_ molecules can be captured, or they could also be the pyrrolic and pyridinic groups where there could be a chemical interaction with the CO_2_ molecules.

**Figure 11:**
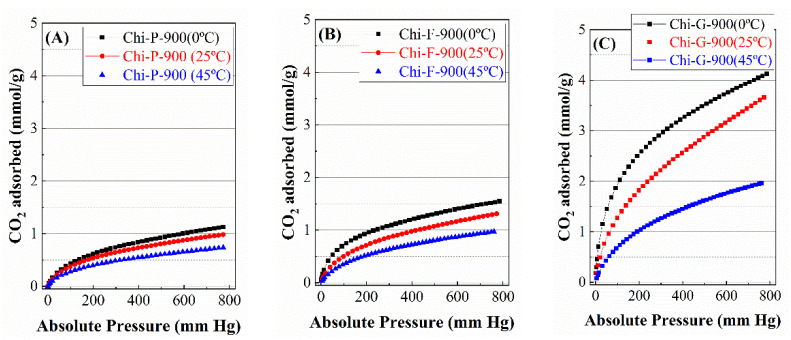
CO_2_ adsorption isotherms at 0–45 °C and 1 bar of the biochars obtained after the pyrolytic treatment of chitosans with different textural properties at 900 °C. (**A**) biochars obtained form chitosan directly, (**B**) biochars obtained from chitosan in the form of film and (**C**) biochars obtained from chitosan in globular form.

Once the CO_2_ adsorption capacity of the biochars at different pyrolysis temperatures have been studied, the next step has is to study the adsorption capacity at different temperatures. For this purpose, adsorbents pyrolyzed at the highest temperature (900 °C) were chosen to carry out this study because the Chi-G-900 sample reached the highest CO_2_ adsorption values (Figure 9). In all cases, it can be observed how an increase in the adsorption temperature worsens the CO_2_ capture capacity [44]. In this sense, it has been reported in the literature that those adsorbents where the adsorption of CO_2_ takes place physically, i.e., the CO_2_ molecules, are retained in highly microporous materials due to the high quadrupole moment of the CO_2_ molecules [45]. Thus, the adsorption study at 45 °C, the temperature at which flue gases are frequently emitted, shows a clear decrease in the CO_2_ adsorption capacity. In the case of commercial chitosan pyrolyzed at 900 °C (Figure 11A), adsorption at 45 °C only attains a CO_2_ adsorption of 0.74 mmol/g at 1 bar of pressure. This value is also low for chitosan in the form of film and subsequently pyrolyzed at 900 °C (Figure 11B), reaching a CO_2_ adsorption of 0.99 mmol/a at 45 °C and 1 bar of pressure. Finally, the chitosan treated with the freeze-drying method and pyrolyzed at 900 °C (Figure 11C) achieves the highest CO_2_ adsorption at this temperature with an adsorption capacity of 1.94 mmol/g at 1 bar of pressure.

Once the adsorption isotherms at different adsorption temperatures were studied, these isotherms were fitted according to the Toth equation [30]. The low ARE values suggest that the isotherms were well-adjusted to the Toth adsorption model.

The adsorption parameters reported in Table 4 show how the adsorbent with the highest CO_2_ uptake values throughout the studied temperature range (Chi-G-900) is also the adsorbent with the higher affinity for the adsorbate, as indicated by the *b*_0,*I*_ values. This stronger affinity is ascribed to the narrower pore diameter, which allows for the retention of higher CO_2_-levels (Table 1). However, the other adsorbents synthesized from chitosan with different textural properties (Chi-P-900 and Chi-F-900) display lower affinity values due to their poorer textural properties. However, it should also be noted that the affinity parameter values decrease when the adsorption temperature increases. From these data, it can be inferred that the adsorption must take place from a physical process, since this type of adsorption is favored at a low temperature and adsorbent with a narrow pore [13,24,25].

The presence of N-species in the form of pyrrolic and pyridinic groups could promote the chemical adsorption of CO_2_ molecules. With this chemical adsorption, increasing the adsorption temperature should improve CO_2_ uptake values; however, these results have not been observed in the present study. Thus, it is expected that the CO_2_ adsorption using biochars obtained from chitosans with different textural properties as adsorbents must be through physical adsorption, as suggested by the narrow pore diameter (Table 1), while the chemical adsorption of CO_2_ must be negligible.

## 5. Conclusions

A commercial chitosan has been modified to give rise to films and globular structures. The pyrolysis of these materials between 500 and 900 °C has generated biochars with different textural properties between them. Thus, it can be observed that the commercial chitosan and the chitosan in the form of a film collapse their structure as the pyrolysis temperature increases. However, chitosan modified by the freeze-drying method seems to be more resistant to pyrolytic treatment, improving its textural properties, even at 900 °C. Under these conditions, the biochar obtained is an amorphous graphite with N-pyridinic and N-pyrrolic groups coming from the -NH_2_ group at the C2 position of the chitosan.

The study of the CO_2_-adsorption capacity shows how the Chi-G-900 sample is the best adsorbent due the high and narrow microporosity, which allows it to host a high proportion of CO_2_ molecules in its framework, obtaining the highest value of 4.11 mmol/g at 0 °C and 1 bar of pressure. In the case of the biochars obtained from the commercial chitosan and the chitosan modified by the freeze-drying method, the CO_2_-adsorption capacity worsens when the pyrolytic treatment takes place at 900 °C, confirming that the framework of both biochars must collapse under this temperature. Finally, the study of the CO_2_-adsorption capacity for Chi-P-900, Chi-F-900 and Chi-G-900 shows how the increase in the adsorption temperature causes a decrease in the CO_2_-adsorption values, suggesting that, despite the presence of N-species, the CO_2_-adsorption must take place from physical interactions.

## Figures and Tables

**Table 1:** Textural properties of biochars determined from CO_2_ adsorption isotherms, as determined using the Dubinin-Astakhov equation [29].

Sample	Micropore Volume (cm^3^/g)	Equivalent Surface Area (m^2^/g)
Chi-P-500	0.122	306
Chi-P-700	0.158	394
Chi-P-900	0.098	232
Chi-F-500	0.100	250
Chi-F-700	0.148	369
Chi-F-900	0.104	257
Chi-G-500	0.155	387
Chi-G-700	0.191	477
Chi-G-900	0.263	657

**Table 2:** Spectral parameters determined by XPS of the biochars obtained after the pyrolytic treatment of chitosans with different textural properties at 900 °C.

Sample	Atomic Concentrations (%)							
	C 1*s*				O 1*s*		N 1*s*	
	C-C	C-O C-N	C=O	O-C=O	O-C=O -C=O	C-OH	Pyrrolic-N	Pyridinic-N
Chi-P-900	62.5	11.5	3.9	2.9	2.0	10.3	1.7	2.1
Chi-F-900	58.2	17.7	6.6	3.4	3.5	5.9	2.2	2.0
Chi-G-900	63.2	17.9	4.3	4.1	2.2	4.2	1.3	2.2

**Table 3:** Toth model parameters for the CO_2_ adsorption of the biochars obtained after the pyrolytic treatment of chitosans with different textural properties at 900 °C (CO_2_ adsorption: 25 °C).

Sample	Parameters				
	q_m0,i_ (mol/kg)	b_0,i_ (1/mm Hg)	Q (J/mol)	t_0,i_	ARE (%)
Chi-P-900	4.80	1.96 × 10^−2^	28,390	0.28	1.14
Chi-F-900	5.34	2.04 × 10^−2^	30,089	0.25	0.97
Chi-G-900	12.12	6.69 × 10^−2^	37.305	0.26	1.31

**Table 4:** Toth model parameters for the CO_2_ adsorption of the biochars obtained after the pyrolytic treatment of chitosans with different textural properties at 900 °C (CO_2_ adsorption: 0–45 °C).

Sample	Parameters				
	q_m0,i_ (mmol/g)	b_0,i_ (1/mm Hg)	Q (KJ/mol)	t_0,i_	ARE (%)
Chi-P-900(0 °C)	5.01	3.55 × 10^−2^	29.155	0.24	1.57
Chi-P-900(25 °C)	4.80	1.96 × 10^−2^	28.390	0.28	1.14
Chi-P-900(45 °C)	2.95	4.97 × 10^−3^	25.115	0.38	2.15
Chi-F-900(0 °C)	6.15	4.15 × 10^−2^	31.155	0.27	1.17
Chi-F-900(25 °C)	5.34	2.04 × 10^−2^	30.089	0.25	0.97
Chi-F-900(45 °C)	3.15	5.99 × 10^−3^	26.487	0.28	1.98
Chi-G-900(0 °C)	14.15	8.15 × 10^−2^	40.151	0.33	2.01
Chi-G-900(25 °C)	12.12	6.69 × 10^−2^	37.305	0.26	1.31
Chi-G-900(45 °C)	7.18	5.15 × 10^−2^	32.156	0.24	1.57

## Data Availability

Data is contained within the article or Supplementary Materials.

## Supplementary Materials

The following supporting information can be downloaded at: https://www.mdpi.com/article/10.3390/polym14235240/s1, Figure S1: Scheme of the solubilization of chitosan under acid pH, Figure S2: SEM images of Chi-P (A), Chi-F (B), Chi-G (C) and their respective samples pyrolyzed at 900 °C: Chi-P-900 (D), Chi-F-900 (E) and Chi-G-900 (F). (Scale: 100 µm).
