# Supplementary material for: Modification of the Textural Properties of Chitosan to Obtain Biochars for CO2-Capture Processes
Source: Polymers (Basel). 2022 Dec 1;14(23):5240. doi: 10.3390/polym14235240 (PMC9739784; doi:10.3390/polym14235240)
Supplement: Supplementary file 1 [file polymers-14-05240-s001.zip › polymers-2034652-supplementary.pdf]

Supplementary information

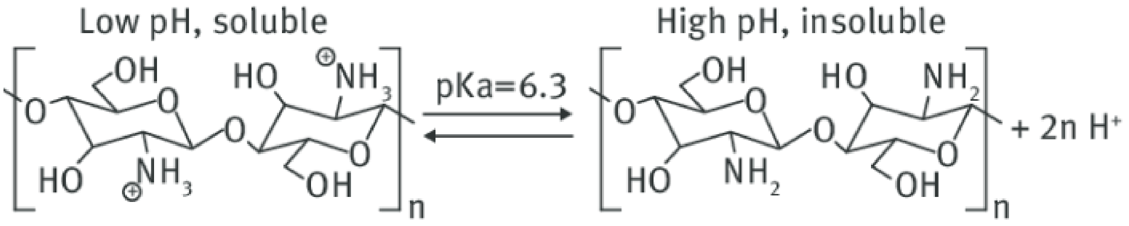

Figure S1. Scheme for the solutibization of chitosan.

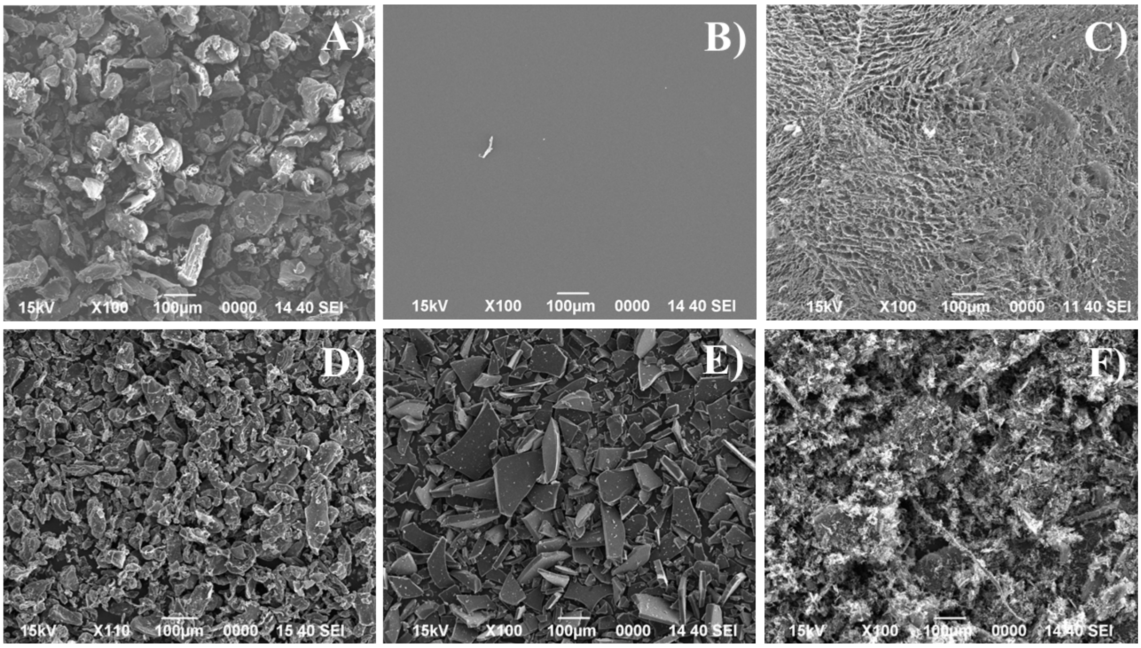

Figure S2. SEM images of Chi-P (A), Chi-F (B), Chi-G (C) and their respective samples pyrolyzed at 900 °C: Chi-P-900 (D), Chi-F-900 (E) and Chi-G-900 (F). (Scale: 100 μm).
